# The Arabic Canadian Occupational Performance Measure (Arabic-COPM) in Pediatric Rehabilitation: Quantitative Reliability Study

**DOI:** 10.2196/87822

**Published:** 2026-10-08

**Authors:** Hikmat Hadoush, Rawan Sweileh, Nihad Almasri

**Affiliations:** 1 Department of Physiotherapy, Faculty of Health Sciences University of Sharjah Sharjah, Sharjah United Arab Emirates; 2 Department of Rehabilitation Sciences, Faculty of Applied Medical Sciences Jordan University of Science and Technology Irbid Jordan; 3 Department of Physiotherapy Qatar University Doha, Baladīyat ad Dawḩah Qatar

**Keywords:** Canadian Occupational Performance Measure, psychometrics, interrater reliability, children with disabilities, family relations

## Abstract

**Background:**

The Canadian Occupational Performance Measure (COPM) has achieved global implementation across 35 countries in more than 20 language versions. However, its psychometric properties for children with physical disabilities remain unverified.

**Objective:**

This study aimed to evaluate the interrater and test-retest reliability of the Arabic version of the COPM (Arabic-COPM) in this pediatric population.

**Methods:**

A cross-sectional study was conducted using a convenience sample of parents of children with physical disabilities recruited from multi-rehabilitation centers. For the interrater reliability assessment, 100 parents completed the Arabic-COPM through independent interviews with 2 raters. A subsample of 30 (30%) parents repeated the assessment after a 14-day interval for the test-retest reliability analysis, in which they identified their children’s 5 most significant daily activity problems and rated the performance and satisfaction levels for each problem using standardized 10-point scales.

**Results:**

Reliability analyses demonstrated excellent psychometric properties. Intraclass correlation coefficients (ICCs) for interrater reliability were 0.84 (performance) and 0.80 (satisfaction), while test-retest reliability coefficients were 0.83 and 0.91, respectively. Interrater agreement analysis revealed 73% concordance in problem prioritization between independent assessors. Bland-Altman plots indicated narrow limits of agreement for both performance (−1.69 to 1.60) and satisfaction scores (−1.03 to 0.98). Cohen κ analysis for problem categorization demonstrated slight-to-fair interrater agreement and slight-to-moderate test-retest agreement across COPM domains. Content analysis identified a predominant concern with daily activities (82%-85% of problems), particularly mobility functions, followed by dressing and eating subcategories.

**Conclusions:**

The Arabic-COPM demonstrated excellent reliability metrics for both interrater consistency and temporal stability when administered to parents of children with physical disabilities. These robust measurement properties support its application as a validated instrument for implementing family-centered rehabilitation approaches in Arabic-speaking clinical contexts.

## Introduction

Pediatric physical disability encompasses a spectrum of nonprogressive developmental conditions, including cerebral palsy and spina bifida, which originate from impairments in the developing nervous system [1]. A primary consequence of these disabilities is a significant limitation in the performance of daily activities, adversely affecting fundamental skills such as transferring, standing, and walking, and ultimately compromising functional independence [2,3]. In contemporary rehabilitation, enhancing functional performance and community participation are central objectives. This aligns with a paradigm shift over recent decades toward a family-centered model of care in pediatric rehabilitation [4,5]. This approach necessitates a direct focus on the challenges faced by parents of children with disabilities [6], particularly concerning the breadth and quality of their children’s performance in daily activities [7]. Within this model, families are integral partners in all phases of service delivery, from goal-setting and treatment planning to evaluating outcomes. The therapist’s role is to be responsive to parental concerns and to equip families with the necessary information for collaborative decision-making [8]. A prominent outcome measure designed to capture this family perspective is the Canadian Occupational Performance Measure (COPM) [9].

The COPM is a semistructured interview developed as a client-centered instrument by Law et al [10]. Its international adoption is evidenced by its availability in over 20 languages across 35 countries [11]. The measure is designed to holistically capture and quantify an individual’s functional performance, roles, and environmental needs, facilitating a process in which clients can freely articulate and explore their problems with a therapist [12]. By eliciting clients’ perceptions of their own performance and their satisfaction with that performance in self-identified areas of concern, the COPM fosters a collaborative partnership. This cooperation ensures the client’s active involvement throughout the evaluation and intervention process [13].

The reliability of the COPM has been investigated in populations with various health conditions [14-16]. Studies have consistently reported excellent interrater reliability, with intraclass correlation coefficients (ICCs) for performance and satisfaction scores ranging from 0.84 to 0.92 in clients with chronic obstructive pulmonary disease [14], psychiatric disorders [15], and stroke [16]. Furthermore, the consistency of problem identification has been demonstrated across different client groups. For instance, 1 study found that 80% of problems prioritized by parents of children with physical disabilities were reidentified in a second interview with a different rater [17]. Similarly, Eyssen et al [18] reported that 66% of problems were consistently prioritized across assessments in patients with neurological disorders.

Despite the expanding use of the COPM in clinical and research settings [8,19-21], the reliability of the Arabic version of the COPM (Arabic-COPM) for parents of children with physical disabilities remains unestablished. Given its importance in family-centered rehabilitation, this study aimed to evaluate the interrater and test-retest reliability of the Arabic-COPM in this population, while also identifying the activity and participation priorities most reported by families.

## Methods

### Study Design and Participants

This study used a cross-sectional, multicenter design to evaluate the reliability of the Arabic-COPM. A convenience sample of 113 parents or primary caregivers of children with physical disabilities was recruited from various rehabilitation centers and societies in Jordan. The final analysis included 100 participants who completed the study protocol, while 13 were excluded due to scheduling conflicts or failure to attend follow-up appointments.

Inclusion criteria were (1) parents or primary caregivers of children diagnosed with physical disabilities stemming from nonprogressive neurological disorders, as confirmed by neurologists; (2) children demonstrating functional limitations in multiple activities of daily living; and (3) parents possessing sufficient proficiency in Modern Standard Arabic to comprehend and respond to interview questions. Exclusion criteria included parents of children with progressive neurological conditions, parents of children with cognitive or communication impairments that would hinder interview participation, and families unable to commit to the study timeline.

### Ethical Considerations

The research protocol received full approval from the institutional review board of King Abdallah University Hospital of Jordan University of Science and Technology (KAUH342020). The study adhered to the principles outlined in the Declaration of Helsinki. Prior to enrollment, all participants engaged in comprehensive briefing sessions detailing the study objectives, procedures, potential risks, and benefits, following which written informed consent was obtained using institution-approved documentation.

### COPM Administration and Assessment Protocol

The COPM was used as the primary client-centered assessment to identify significant challenges in the children’s daily functioning. Problem areas identified by families were categorized using a standardized coding system developed by Pollock and Stewart [22], which organizes priorities into 3 main domains: daily activities (with subcategories of self-care, mobility, and communication), productivity (including household management, school participation, and community engagement), and leisure (encompassing socialization, community entertainment, quiet recreation, and physical recreation). The self-care domain was further broken down into 5 specific components: eating, dressing, bathing, toileting, and hygiene.

### Standardized Administration Protocol

All assessments were conducted by two independent licensed clinical rehabilitation therapists, each assessing participants independently with experience in pediatric rehabilitation and prior training in the administration of the COPM. Both raters underwent standardized calibration sessions before data collection to ensure consistency in interview administration, scoring procedures, and problem categorization. The administration followed a structured five-step [22] process using the validated Arabic translation of the COPM. The first step was problem identification: parents enumerated activities and tasks presenting significant challenges in their child’s daily routine. The second step was importance scaling: using a 10-point metric (1=“minimal importance” to 10=“critical importance”), parents rated all identified concerns. The third step was priority selection: through facilitated discussion, parents distilled their concerns to 5 paramount issues. The fourth step was performance evaluation: each prioritized activity received a performance rating (1=“complete inability” to 10=“optimal performance”). The fifth step was satisfaction evaluation: parents rated their satisfaction with their child’s performance or each problem (1=“not satisfied” to 10=“extremely satisfied”). Mean scores for performance and satisfaction were calculated by averaging the ratings across the 5 prioritized problems.

### Reliability Testing Framework

Although the COPM is classified as a patient-reported outcome measure (PROM), it differs from conventional self-administered PROMs because it is conducted through a semistructured clinician-led interview rather than a fixed questionnaire completed independently by respondents. During COPM administration, the clinician actively facilitates problem identification, clarification, prioritization, and scoring of performance and satisfaction. Therefore, the interrater reliability assessment is considered crucial and relevant, as it has also been evaluated in previous COPM validation studies to determine the consistency of identified priorities and scores across independent assessors.

A multiphase protocol was implemented to assess reliability. To evaluate interrater reliability, 100 parents completed the Arabic-COPM twice on the same day, administered by 2 independent raters who were blinded to each other’s findings. For test-retest reliability, a random subsample of 30 (30%) parents repeated the assessment with the same rater after a 14-day interval, following an identical administration procedure (Figure 1).

**Figure 1:**
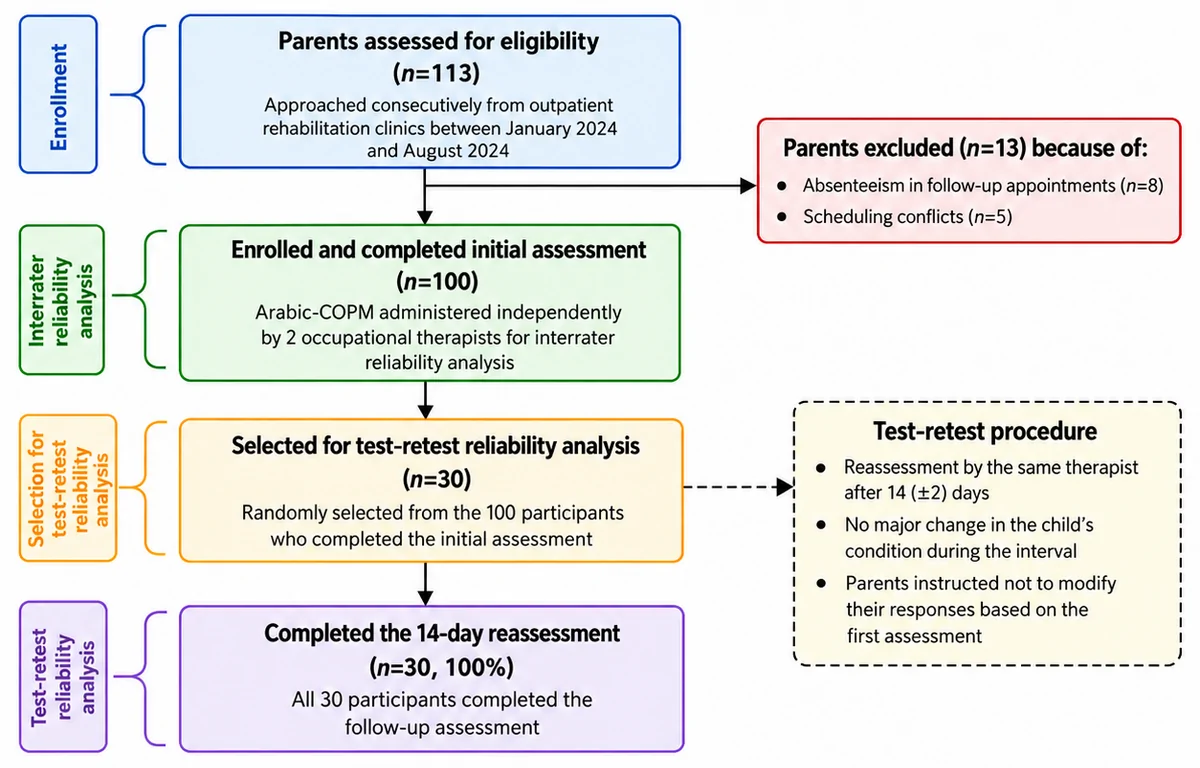
Study flowchart. COPM: Canadian Occupational Performance Measure.

### Statistical Analysis

The psychometric evaluation of the Arabic-COPM incorporated comprehensive reliability and agreement analyses. Reliability was assessed using ICCs with a 2-way mixed-effects model, applying absolute agreement for interrater comparisons and consistency for test-retest analyses [23]. Interpretation of reliability coefficients was performed cautiously and in the context of previous COPM and rehabilitation reliability studies, in which the interpretation followed established benchmarks of 0.40 to 0.59 (fair), 0.60 to 0.74 (good), and >0.75 (excellent). However, categorical thresholds for ICC interpretation may vary across methodological frameworks and should not be considered absolute indicators of measurement quality.

Agreement assessment used multiple complementary approaches. For quantitative agreement, Bland-Altman methodology was implemented to evaluate measurement concordance [24]. This involved plotting differences between paired measurements against their means, with calculation of mean bias (d̄) and 95% limits of agreement (LOA; LOA=d̄±1.96×SD) [25]. To address visualization challenges in datasets with restricted ranges, traditional scatterplots were enhanced through hexagonal binning techniques that aggregate observations into density-based visualizations, providing better pattern recognition than conventional displays.

For qualitative agreement, problem prioritization consistency was assessed by calculating the proportion of first rater–identified problems that were similarly prioritized by the second rater. Additionally, Cohen κ coefficient was computed to evaluate interrater agreement across COPM domains (daily activity, productivity, and leisure) [26], with interpretation standards: <0.00 (none), 0.00 to 0.20 (slight), 0.21 to 0.40 (fair), 0.41 to 0.60 (moderate), 0.61 to 0.80 (substantial), and 0.81 to 1.00 (almost perfect agreement) [27].

All analyses were conducted using Python (version 3.12; Python Software Foundation; *Matplotlib*) with statistical significance set at *P*<.05, maintaining methodological consistency across comparisons in accordance with contemporary quantitative method evaluation standards.

## Results

### Sample Characteristics

The final sample consisted of 100 parents of children with physical disabilities. The children had a mean age of 5.4 (SD 2.7) years, with a near-even sex distribution (54 male children and 46 female children). The majority of respondents (n=83, 83%) were the children’s mothers. Detailed demographic characteristics are presented in Table 1.

**Table 1:** Demographic characteristics of the parents and their children with physical disabilities.

Characteristics				Participants
**Children’s characteristics (n=100)**				
	Age (years), mean (SD)		5.4 (2.7)	
	Weight (kg), mean (SD)		17.6 (6.8)	
	Height (cm), mean (SD)		98.3 (19.5)	
	**Diagnosis, n (%)**			
		Cerebral palsy	60 (60)	
		Spina bifida	20 (20)	
		Other diagnoses	20 (20)	
	**Sex, n (%)**			
		Male	54 (54)	
		Female	46 (46)	
**Parents’ characteristics (n=100), n (%)**				
	**Relationship with the child**			
		Mother	83 (83)	
		Father	10 (10)	
		Other	7 (7)	
	**Educational level**			
		University degree	57 (57)	
		Nonuniversity degree	43 (43)	
	**Monthly income**			
		High income (US >$900)	24 (24)	
		Middle income (US $450-$900)	56 (56)	
		Low income (US <$450)	20 (20)	

### Interrater Reliability and Agreement

The Arabic-COPM demonstrated excellent interrater reliability. The ICCs for the mean scores were 0.84 (95% CI 0.77-0.89; *P*<.001) for performance and 0.80 (95% CI 0.70-0.86; *P*<.001) for satisfaction.

Regarding qualitative agreement on problem identification, the first and second raters identified 460 and 450 problems, respectively. A high level of concordance was observed, with 338 (73.5%) of the 460 problems prioritized by the first rater being similarly prioritized by the second rater (Table 2). On the other hand, Cohen κ analysis across the COPM main categories (daily activity, productivity, and leisure) demonstrated slight-to-fair interrater agreement.

**Table 2:** Distribution of parent-prioritized problems identified during the Arabic version of the Canadian Occupational Performance Measure (COPM) assessment, categorized by main categories, subcategories, and self-care subcomponents, by raters^a^.

Categories and subcategories				Rater R, n (%)		Rater S, n (%)		Comparison (percentage points)
**Daily activity**				380 (82)		383 (85)		+3
	Mobility		226 (49)		225 (50)		+1	
	**Self-care**		150 (33)		154 (34)		+1	
		Eating	38 (25)		34 (22)		−3	
		Dressing	53 (35)		53 (34)		−1	
		Toileting	22 (15)		33 (21)		+6	
		Bathing	29 (19)		22 (14)		−5	
		Hygiene	9 (6)		12 (8)		+2	
		Communication	4 (1)		4 (1)		0	
**Productivity**				30 (7)		24 (5)		−2
	School		30 (7)		24 (5)		−2	
	Household		0 (0)		0 (0)		0	
	Community		0 (0)		0 (0)		0	
**Leisure**				50 (11)		43 (10)		−1
	Socialization		14 (3)		16 (4)		+1	
	Community entertainment		13 (3)		10 (2)		−1	
	Quiet recreation		1 (1)		0 (0)		−1	
	Physical recreation		22 (5)		17 (4)		−1	

^a^This table summarizes all problems identified by parents during the COPM interview and classified using the standardized COPM coding system. The 3 main categories (daily activity, productivity, and leisure) and their corresponding subcategories are presented alongside detailed self-care subcomponents. For each classification level, the table reports the number and percentage of problems identified independently by rater R and rater S. The comparison column (percentage points) reflects the proportional difference between raters for each subcategory, providing an integrated view of agreement patterns across the full activity and participation domain. Rater R and Rater S were 2 independent raters.

Regarding quantitative interrater agreement, Bland-Altman analysis with hexagonal binning visualization demonstrated strong quantitative agreement between raters for both performance and satisfaction scores. The LOA were narrow, ranging from −1.69 to 1.60 (mean bias −0.048, SD 0.84) for performance scores and from −1.03 to 0.98 (mean bias −0.024, SD 0.51) for satisfaction scores. The corresponding Bland-Altman plots (Figures 2 and 3), enhanced through hexagonal binning to visualize data density, show that most observations were clustered around the mean bias line and fell within the 95% LOA.

Supporting these findings, linear regression analysis revealed no evidence of proportional bias. The relationship between score differences and mean scores was nonsignificant for both performance (*F*_1,98_=2.80; *P*=.10; *R*^2^=0.028; Figure 2) and satisfaction scores (*F*_1,98_=0.83; *P*=.36; *R*^2^=0.008; Figure 3), indicating consistent agreement across the measurement scale.

**Figure 2:**
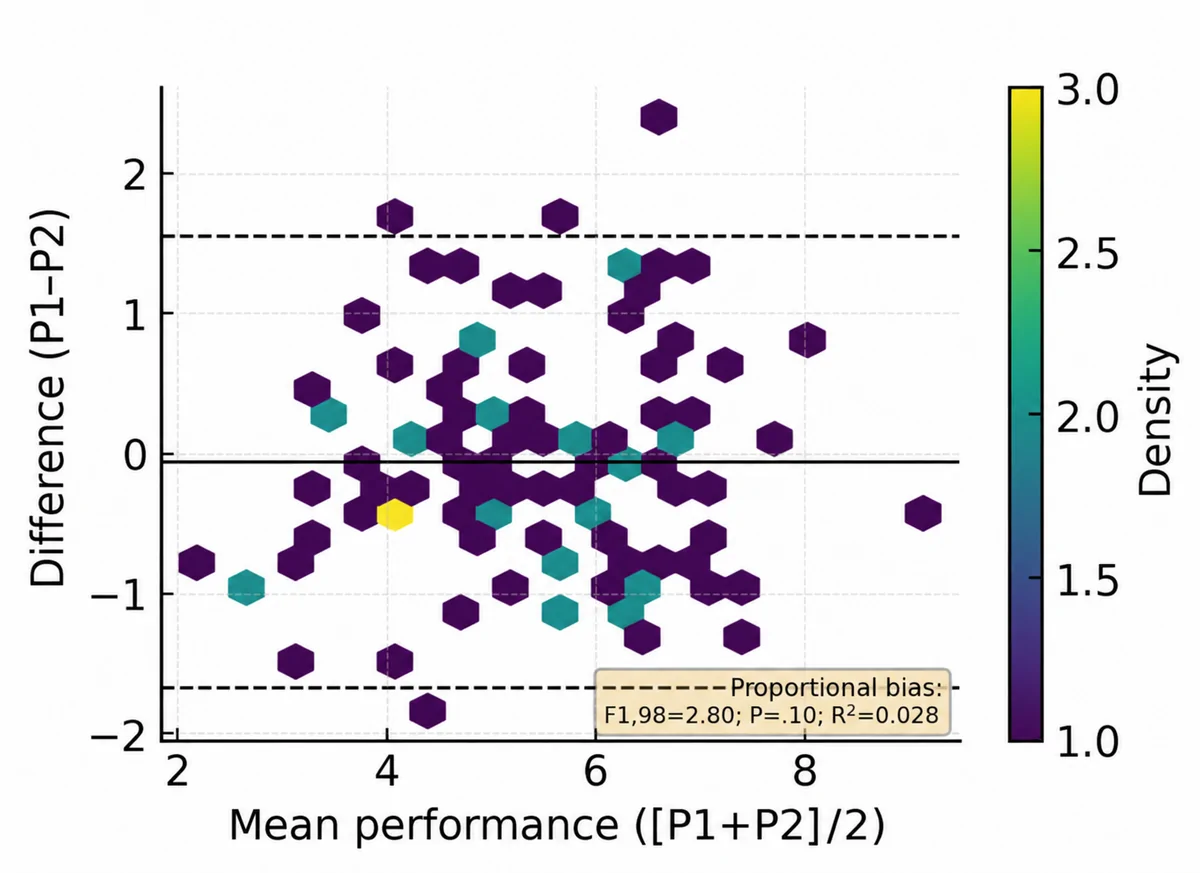
Hexbin Bland-Altman plot of interrater agreement for Canadian Occupational Performance Measure (COPM) performance scores, with proportional bias analysis. This Hexbin Bland-Altman analytical plot illustrates the agreement between 2 independent raters for COPM performance scores. The mean of the 2 raters’ scores is plotted on the x-axis, and the score difference (P1−P2) is plotted on the y-axis. Each hexagonal cell represents the local density of overlapping observations, improving clarity in regions of high point concentration. The solid horizontal line represents the mean bias, while the dashed lines indicate the 95% limits of agreement. Proportional bias was examined using linear regression of score differences on their mean values. The association was nonsignificant (F1,98=2.80; *P*=.10; R2=0.028), demonstrating no systematic proportional bias.

**Figure 3:**
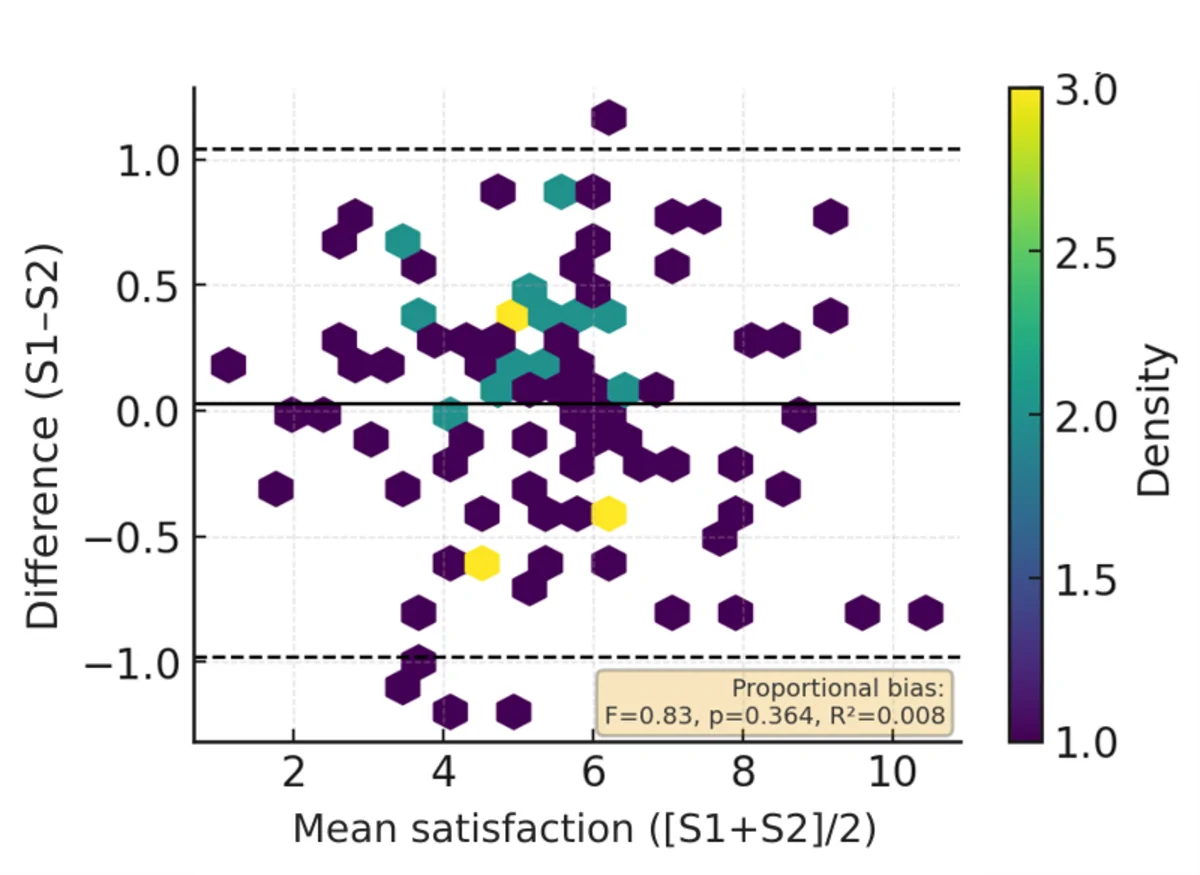
Hexbin Bland-Altman plot of interrater agreement for Canadian Occupational Performance Measure (COPM) satisfaction scores, with proportional bias analysis. This Hexbin Bland-Altman plot presents the interrater agreement for COPM satisfaction scores. The x-axis shows the mean satisfaction rating between raters, and the y-axis represents the difference (S1−S2). Hexagonal density shading highlights regions where overlapping values occur. The solid line depicts the mean bias, and the dashed lines represent the 95% limits of agreement. Proportional bias was examined using linear regression of score differences on their mean values. The association was nonsignificant (F1,98=0.83; *P*=.36; R2=0.008), demonstrating no systematic proportional bias.

### Test-Retest Reliability and Agreement

The Arabic-COPM demonstrated excellent temporal stability, with test-retest reliability ICCs of 0.83 (95% CI 0.64-0.92; *P*<.001) for performance scores and 0.91 (95% CI 0.81-0.95; *P*<.001) for satisfaction scores.

In terms of test-retest quantitative agreement, Bland-Altman analysis, visualized through hexagonal binning plots (Figures 4 and 5), demonstrated strong quantitative agreement across the 2-week interval. The LOA were narrow, spanning from −1.43 to 1.51 (mean bias 0.04, SD 0.75) for performance scores and from −0.56 to 0.78 (mean bias 0.11, SD 0.34) for satisfaction scores. The density-based plots show a tight clustering of data points within the LOA, indicating minimal systematic drift and high measurement repeatability over time.

On the other hand, Cohen κ analysis across the COPM main categories (daily activity, productivity, and leisure) demonstrated slight-to-fair interrater agreement.

**Figure 4:**
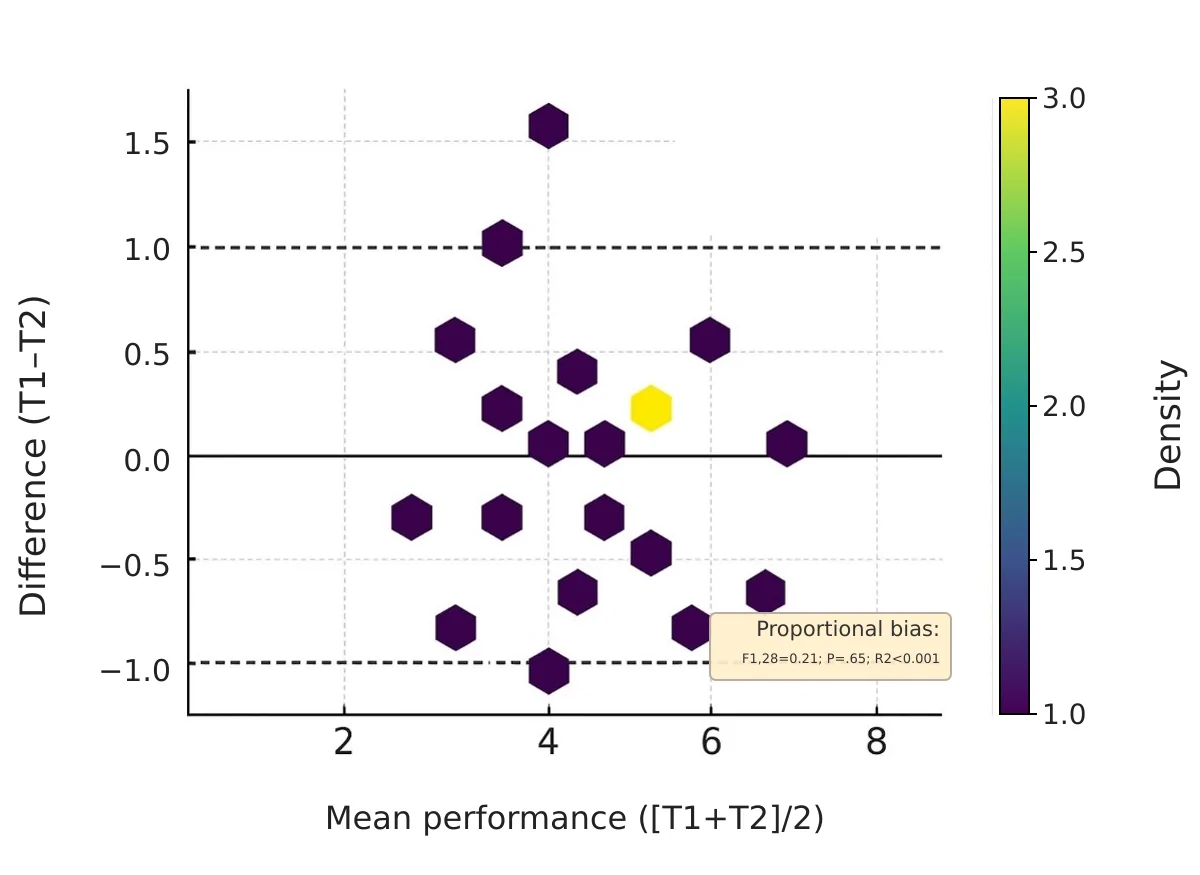
Hexbin Bland-Altman plot of test-retest agreement for Canadian Occupational Performance Measure (COPM) performance scores (2-week interval), with proportional bias assessment. This Hexbin Bland-Altman analytical plot illustrates the test-retest agreement of COPM performance scores obtained from the same assessor over a 2-week interval. The mean of the 2 performance scores (T1 and T2) is plotted on the x-axis, and their difference (T1−T2) on the y-axis. Density-based hexagonal binning highlights regions of overlapping values, improving interpretability relative to conventional scatter plots. The mean bias is represented by the solid horizontal line, with the 95% limits of agreement shown as dashed lines. Regression analysis demonstrated that the relationship between score differences and their means was nonsignificant, indicating no proportional bias.

**Figure 5:**
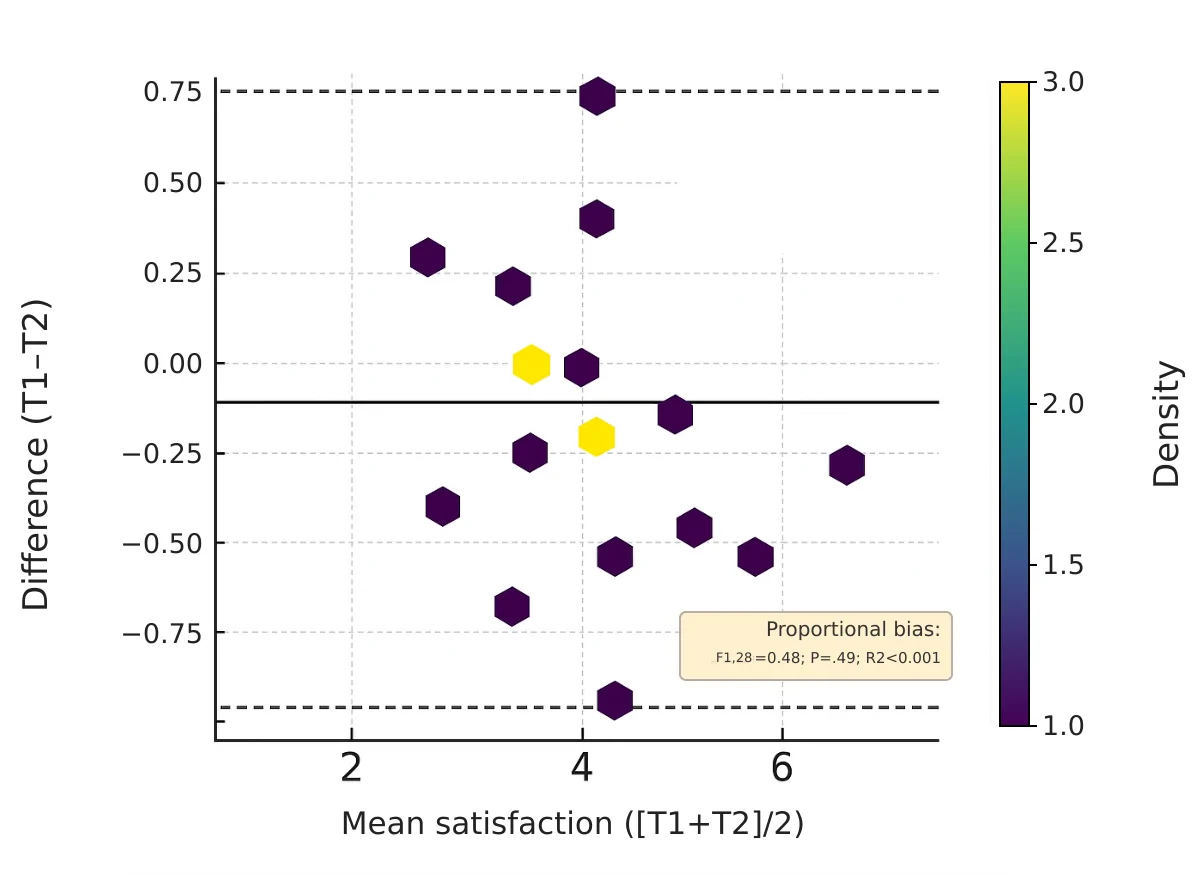
Hexbin Bland-Altman plot of test-retest agreement for Canadian Occupational Performance Measure (COPM) satisfaction scores (2-week interval), with proportional bias assessment. This hexbin Bland-Altman plot displays the test-retest reliability of COPM satisfaction scores across a 2-week interval. The mean satisfaction score is presented on the x-axis, and the difference between sessions (T1−T2) on the y-axis. Density-based hexagonal binning highlights regions of overlapping values, improving interpretability relative to conventional scatter plots. The mean bias is represented by the solid horizontal line, with the 95% limits of agreement shown as dashed lines. Regression analysis demonstrated that the relationship between score differences and their means was nonsignificant, indicating no proportional bias.

### Families’ Prioritized Concerns

Across both raters, a total of 460 problems were identified by rater R and 450 by rater S (Table 2). The vast majority of parent-prioritized problems fell within the daily activity main category, accounting for 82% to 85% of all reported concerns. Within daily activity, parents expressed the greatest concern for the mobility subcategory, which represented approximately 50% of all identified problems for both raters.

The second most frequently reported main category was leisure (10%-11%), with parents primarily emphasizing difficulties in the physical recreation subcategory (≈5% of all problems). In contrast, productivity concerns were least commonly reported (5%-7%) and were almost exclusively related to the school subcategory, with no meaningful concerns in household or community tasks.

Within the daily activity category, the self-care subcategory represented a substantial proportion of the identified priorities. When examining self-care in more detail, parents reported the highest concern for dressing, followed by eating, with additional—but comparatively fewer—concerns related to toileting, bathing, and hygiene.

## Discussion

This study evaluated the interrater and test-retest reliability of the Arabic-COPM in parents of children with physical disabilities. The findings demonstrated strong reliability across both performance and satisfaction scores, supporting the consistency and stability of the Arabic-COPM within pediatric rehabilitation settings.

### Psychometric Strength and Cross-Cultural Consistency

The Arabic-COPM exhibited excellent reliability metrics that meet or exceed established standards for outcome measures in rehabilitation. The high ICCs ranging from 0.80 to 0.91 across both interrater and test-retest evaluations indicate remarkable consistency between different clinicians and stability over time. Particularly noteworthy is the exceptional temporal reliability of satisfaction scores (ICC=0.91), suggesting that parental satisfaction with their child’s performance represents a particularly stable construct that can be reliably tracked throughout the rehabilitation process.

These reliability findings are generally consistent with several previous COPM studies conducted across different clinical populations and cultural contexts. Similar ICC ranges have been reported in patients with chronic obstructive pulmonary disease (approximately 0.90) [14], psychiatric disorders (approximately 0.85) [15], and ankylosing spondylitis (approximately 0.92) [13], supporting the stability of the COPM across diverse rehabilitation settings.

However, reliability findings in the broader COPM literature have not been entirely uniform. Some studies and reviews [28,29] have reported lower or more variable agreement levels depending on the assessed population, administration procedures, and methodological approaches. Such variability may reflect the individualized and semistructured nature of the COPM, in which participant priorities, interviewer interaction, contextual influences, and clinical interpretation can affect scoring consistency. Therefore, the findings of the present study should be interpreted within the context of the heterogeneous reliability literature surrounding the COPM.

These reliability findings demonstrate remarkable consistency with previous COPM validation studies across diverse populations and cultural contexts. The similarity of our ICC values to those reported in patients with chronic obstructive pulmonary disease (approximately 0.90) [14], psychotic disorders (approximately 0.85) [15], and ankylosing spondylitis (approximately 0.92) [13] suggests that the core client-centered methodology of the COPM maintains its measurement properties across linguistic and cultural adaptations. This cross-cultural consistency reinforces the fundamental robustness of the COPM framework while simultaneously validating the quality of the Arabic adaptation.

### Comprehensive Agreement Analysis

Beyond conventional reliability coefficients, our implementation of multiple agreement methodologies provides a more nuanced understanding of the measure’s performance. The Bland-Altman analyses with hexagonal binning visualization revealed narrow LOA with minimal systematic bias, demonstrating precise measurement characteristics at the individual score level. The 73.5% concordance in problem prioritization between independent raters indicates a moderate level of agreement between assessors. Although this finding supports a reasonable degree of consistency in the Arabic-COPM administration process, the remaining variability highlights the influence of interviewer interaction and individualized problem prioritization inherent in semistructured client-centered assessments.

When contextualized within the broader COPM literature, our interrater agreement rate of 73.5% falls between the agreement rates reported in other populations, reflecting the inherent balance between standardization and individualization in client-centered measures. This rate closely approximates the 80% agreement reported in similar pediatric populations [17] while substantially exceeding the 56% and 66% rates reported in stroke and mixed neurological populations [16,18]. This gradient likely reflects differences in cognitive clarity, goal stability, and the nature of therapeutic relationships across these diverse clinical populations.

### Therapeutic Dialogue and Clinical Implementation

The variability observed in qualitative agreement measures should not be interpreted as evidence against standardization or measurement consistency. The COPM combines standardized scoring procedures with a semistructured individualized interview format. Although individualized problem identification and interviewer interaction may introduce some variation in prioritized responses across assessors, the strong quantitative reliability findings observed in the present study suggest that the Arabic-COPM maintains acceptable measurement consistency within a structured client-centered assessment framework.

### Cultural and Developmental Priorities in Pediatric Rehabilitation

The pattern of problem prioritization revealed important insights into the concerns of Arabic-speaking families of children with physical disabilities. The overwhelming focus on daily activities (82%-85%), particularly mobility and self-care functions such as dressing and eating, provides empirical support for Maslow hierarchy of needs within this specific cultural and clinical context [30]. As Duncan and Blugis [31] articulated in their pediatric adaptation of this framework, families naturally prioritize fundamental needs related to nourishment, personal care, and basic functioning before addressing higher-order productivity or complex leisure pursuits.

This prioritization pattern reflects both practical caregiving realities and developmental aspirations. Parents intuitively recognize that mastery of basic mobility and self-care skills not only reduces caregiver burden but also serves as the essential foundation for peer socialization, educational participation, and eventual transition toward independent living. The predominance of mobility and self-care concerns identified in the present study suggests that families primarily prioritize essential daily functioning needs when identifying rehabilitation goals for children with physical disabilities. In contrast, productivity-related concerns were less frequently reported. These findings may reflect the central importance of functional independence in early pediatric rehabilitation and emphasize the practical relevance of addressing basic daily activities within family-centered rehabilitation planning.

The consistency of these findings with previous research involving children with cerebral palsy [32] suggests that these fundamental concerns may transcend specific diagnostic categories within the broader population of children with physical disabilities.

### Limitations and Future Directions

Although this study establishes the fundamental reliability of the Arabic-COPM, several limitations warrant consideration. The scope of this validation study did not permit exploration of how problem prioritization might vary according to disability severity, socioeconomic factors, or cultural subgroups within the Arabic-speaking population. Another limitation is that absolute reliability indices such as the SE of measurement were not evaluated in the present study. Additionally, CIs for descriptive percentage estimates were not calculated and should be considered in future psychometric studies. Future research should investigate these potential moderating variables to provide more nuanced guidance for clinical implementation. Additionally, the focus on reliability represents only the first step in comprehensive instrument validation. Subsequent studies should establish the responsiveness, minimal clinically important differences, and convergent validity of the Arabic-COPM to fully characterize its measurement properties.

### Clinical Implications and Conclusions

Notwithstanding these limitations, our findings provide strong evidence supporting the integration of the Arabic-COPM into clinical practice throughout the Arabic-speaking world. The measure offers rehabilitation professionals a reliable, culturally adapted tool to authentically engage families in the goal-setting process. The consistent prioritization of basic daily activities underscores the importance of addressing these fundamental concerns as the foundation of any comprehensive rehabilitation program.

In conclusion, the Arabic-COPM demonstrates excellent reliability and clinical utility for implementing family-centered care in pediatric rehabilitation. Its successful validation opens new possibilities for standardized outcome measurement and culturally responsive service delivery across Arabic-speaking communities, ultimately supporting improved rehabilitation outcomes for children with physical disabilities and their families.

## Data Availability

The datasets generated and analyzed during the current study are not publicly available due to ethical and privacy restrictions involving pediatric clinical data and family-reported information. Deidentified data may be made available by the corresponding author upon reasonable request.

## Abbreviations

Arabic-COPM: Arabic version of the Canadian Occupational Performance Measure
COPM: Canadian Occupational Performance Measure
ICC: intraclass correlation coefficient
LOA: limits of agreement
PROM: patient-reported outcome measure
